# Biotin-Avidin-Mediated Capture of Microspheres on Polymer Fibers

**DOI:** 10.3390/molecules24112036

**Published:** 2019-05-28

**Authors:** Lucas Ahrens, Daniel Vonwil, Neha Arya, Aurelien Forget, V. Prasad Shastri

**Affiliations:** 1 Institute for Macromolecular Chemistry, University of Freiburg, 79104 Freiburg, Germany; lucas.aj.ahrens@gmail.com (L.A.); vonwilda@gmail.com (D.V.); neha.arya@gmail.com (N.A.); aurelien.forget@makro.uni-freiburg.de (A.F.); 2 BIOSS Centre for Biological Signalling Studies, University of Freiburg, 79104 Freiburg, Germany; 3 Department of Biochemistry, All India Institute of Medical Sciences Bhopal, Saket Nagar, Bhopal 462020, India

**Keywords:** avidin, biotin, bioreactor, polymer mesh, particle capture, biorthogonal

## Abstract

Systems for efficient and selective capture of micro-scale objects and structures have application in many areas and are of particular relevance for selective isolation of mammalian cells. Systems for the latter should also not interfere with the biology of the cells. This study demonstrates the capture of microspheres through orthogonal coupling using biotin (ligand) and (strept)avidin (receptor). Fibrous poly(ethylene terephthalate) (PET) meshes were hydrolyzed under controlled alkaline conditions to obtain activated surfaces with COOH groups allowing for the functionalization of the PET with biotin of various spacer length. The system capture efficiency was optimized by varying the length of spacer presenting the biotin against streptavidin. In a proof of concept experiment, avidin-functionalized microspheres were used as surrogates for cells, and their capture under dynamic conditions including virous mixing and high-flow rate perfusion is demonstrated. Functionalization of PET meshes with biotin conjugated to longest spacer yielded the most efficient capture of microspheres. These preliminary results lay the foundation for the development of biosystems for capture of specific cells under physiologically relevant conditions, using biorthogonal avidin-biotin interactions.

## 1. Introduction

Selective capture of micrometer-sized objects in solution has many biological applications, from the capture of circulating tumor cells for cancer diagnosis [1], selection of low abundance cells in culture [2] to the capture of a specific cell population in suspension [3]. Several physical and biochemical strategies have been described for the capture of moving cells or microspheres. While physical methods such as filtration [4] or acoustic waves can be used to select cells based on their size [5], biochemical methods such as cell-binding to peptide-functionalized surfaces [6], or immunosorting techniques using magnetic beads [7] allow for the capture of cells based on their biological properties. However, biochemical strategies might affect the cell behavior upon capture, since they are based on the biology of captured cells. Therefore, capture methods relying on biorthogonal binding—that can occur inside of living systems without interfering with essential biochemical processes—offer an approach that does not interfere with the biology of the cells, and are not expected to influence the behavior of the captured cells.

Efficiency of the capturing methods for micrometer-size objects relies on several parameters including (i) surface area in contact with the target object, (ii) density of the capturing motif on a surface, and (iii) nature of interaction between the capturing motif and the microscopic object (covalent, ionic, H-bonding, van der Waals), and (iv) time scale of interaction. Therefore, meshes that offer a high surface area through a dense fiber network are of particular interest as they allow for more substantial surface contact with the objects to capture, and such networks can disturb fluid flow and induce turbulence. In addition to high surface area, capturing surfaces need to have a chemistry allowing for a high-density functionalization of the surface with the capturing motif. Finally, the capturing motif needs to form a strong bond with the micrometer-sized object, be it microsphere or cell.

Poly(ethylene terephthalate) (PET) meshes are extensively used in surgery as abdominal implants [8], or hernia repairs [9]. PET has many benefits in terms of stability and biocompatibility, and its inert surface can be hydrolyzed under mild conditions to yield a carboxylic acid (COOH) activated surface [10]. The COOH groups can then be used to functionalize the PET mesh with a strong capturing motif. In this study, we used a biotin-(strept)avidin protein coupling system as the capture model system. Avidin protein is expressed by avian and amphibians but not in mammals [11,12,13], thus it can be used without interfering with the biology of the human cell. Biotin-(strep)avidin coupling is known as one of the strongest non-covalent biocompatible bonds with a dissociation constant of $10^{-15}$ M in the range of covalent bonds. Once formed, the complex of avidin (receptor analog) with biotin (ligand) is stable, resists changes in pH, and subsequent washing steps [14]. Furthermore, cells can be modified to express the avidin protein on the cell surface, which can be used for targeting biotinylated compounds to a specific tissue [15], or to improve cell attachment on synthetic biotinylated surfaces [16].

In this study, we developed a fibrous mesh capable of capturing microspheres under turbulent flow as a model system. As a capture substrate, we used a surgical non-woven PET mesh offering a high surface area. This mesh was then chemically modified to bare COOH functional groups amenable to further modification. The activated PET mesh was further functionalized with biotin. As a proof of concept, 10 µm diameter avidin-coated microspheres were used to simulate mammalian cell capture. The surface chemistry of the PET mesh was optimized to allow maximum capture of the microspheres. Then, the length of the spacer between the biotin and the PET mesh was varied to identify the optimal distance between biotin and PET surface for the capture of avidin-coated microspheres. The efficacy of the biotinylated PET meshes to capture these microspheres was characterized under non-static conditions. First, the microsphere retention was studied under turbulent mixing and then under perfusion using an in-house developed perfusion bioreactor. These experiments demonstrate the possibility to capture avidin objects within a biotinylated mesh under flow conditions, thus paving the way towards the biorthogonal capture of circulating cells.

## 2. Results

### 2.1. Chemical Activation of Poly(ethylene terephthalate) Meshes

**Figure 1:**
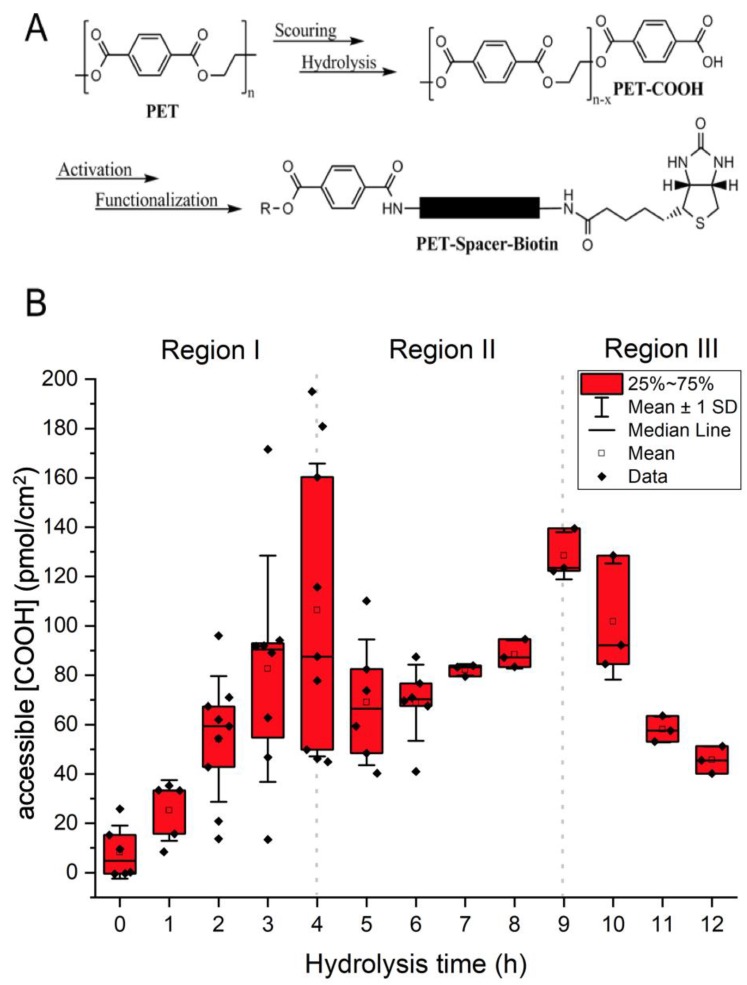
Alkaline hydrolysis of PET meshes. (**A**) Reaction scheme describing PET hydrolysis. (**B**) Sulfo-SDTB quantification of accessible carboxylic acid group concentration on PET meshes with hydrolysis duration. Data are presented as mean ± SD.

Poly(ethylene terephthalate) (PET) can be hydrolyzed to bear carboxylic acid functional groups without impacting the integrity of the fibers (Figure 1A). Following a procedure described by Bide et al. [10], non-woven PET meshes were hydrolyzed with a 0.5% (*w*/*v*) NaOH solution at 98 °C and the amount of surface accessible COOH generated over the 12 hrs of reaction was quantified as a function of time using a colorimetric assay based on sulfosuccinimidyl-4-*O*-(4,4-dimethoxytrityl) butyrate (sulfo-SDTB) [17]. Based on the duration of hydrolysis, three different regions with distinct changes in the rate and extent of accessible COOH concentration were identified (Figure 1B). The first region depicts the first 4 h of hydrolysis, in which a linear increase (R^2^ = 0.98) of accessible COOH was observed that peaked at 106 ± 59 pmol/cm^2^. In the second region (4 to 9 h), a drop in accessible COOH concentration to 69 ± 15 pmol/cm^2^ was observed before it reached another maximum of 128 ± 10 pmol/cm^2^. The last region (9 to 12 h) demonstrated a further drop in concentration to 46 ± 6 pmol/cm^2^.

**Figure 2:**
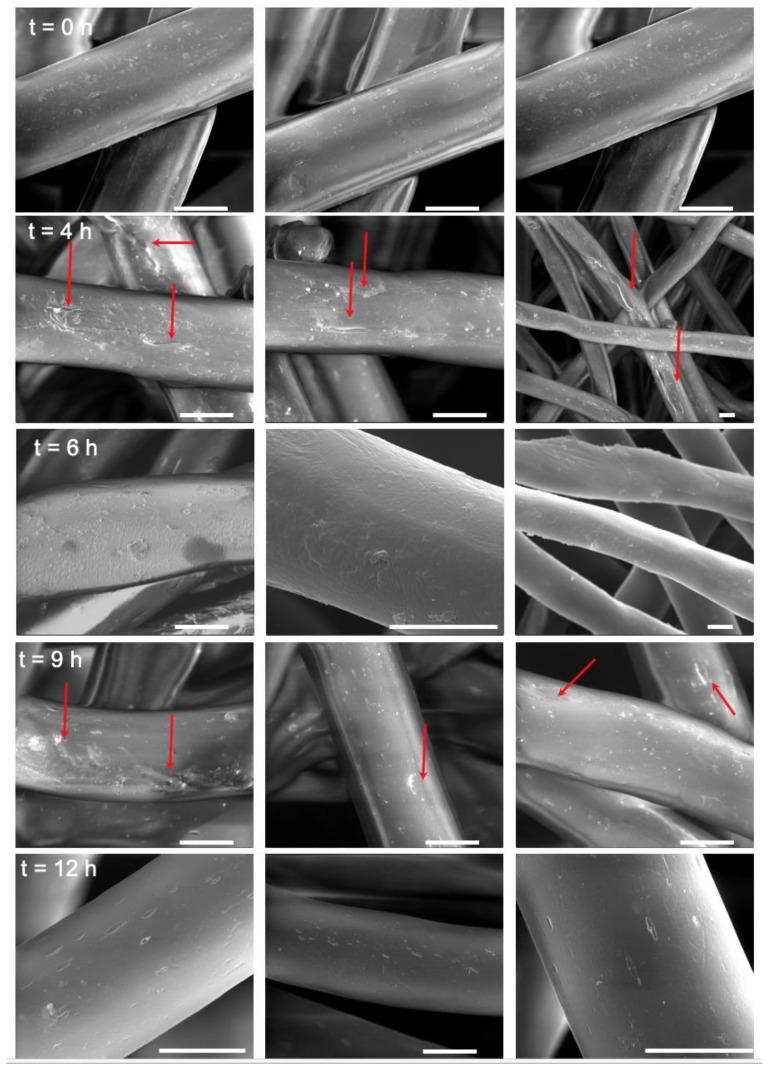
Scanning electron micrographs of representative PET mesh fibers after 0, 4, 6 and 9 h of hydrolysis. Red arrows show surface changes on the PET fibers. Scale bar 10 µm.

To understand the variations in accessible COOH concentration, we imaged the unhydrolyzed PET mesh (0 h) and compared it with the PET mesh after 4 h (region I), 6 h (region II) and 9 h (region III) of hydrolysis (Figure 2). The untreated PET fiber showed a smooth surface. In contrast, PET meshes at the end of 4 h, and 6 h revealed a rough surface. At the end of 9 h, the fibers surface morphology resulted in surface pitting. While the highest accessible COOH concentration was observed after 9 h of hydrolysis, this extended reaction time was accompanied by pitting of the fiber surface. Since we wanted to minimize the changes to the surface morphology of the fibers while having a highly accessible COOH concentration, for further downstream experiments, the hydrolysis time for all further experiments was set to 3 h. This time yielded a concentration of 83 ± 46 pmol/cm^2^ corresponding to 4.9 × 10^13^ accessible COOH groups per cm^2^.

### 2.2. Functionalization of PET Meshes, Binding to Avidin

The COOH-activated PET meshes were functionalized with biotin, a bioorthogonal capturing moiety. For this, COOH was activated with 1-ethyl-3-(3-dimethylaminopropyl)carbodiimide (EDC) in 2-(*N*-morpholino)ethanesulfonic acid (MES) buffer and then transformed into active esters using N-hydroxysuccinimide (NHS) in PBS buffer using a previous protocol (Figure 1A) [18,19,20,21].

**Figure 3:**
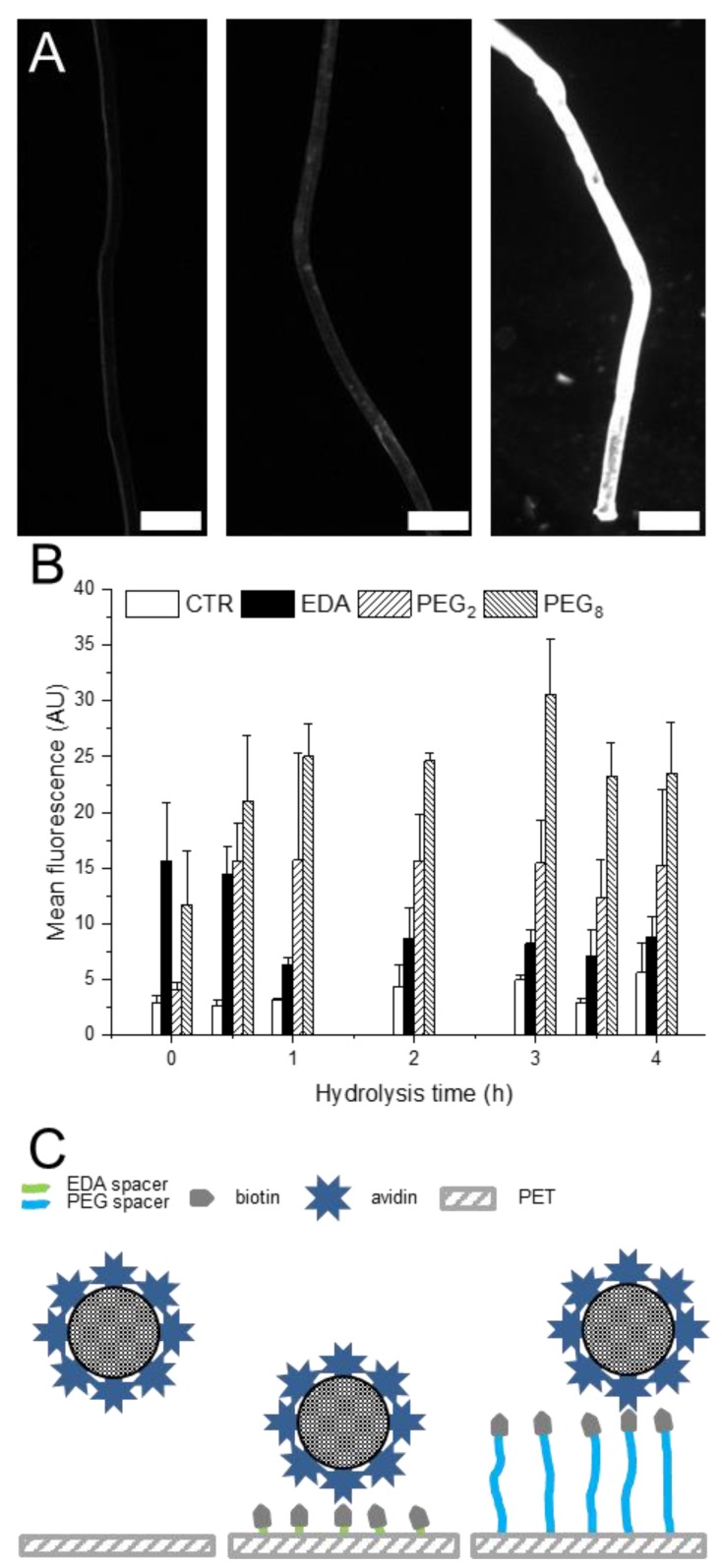
Effect of spacer length on avidin binding. (**A**) Fluorescence images of PET fibers treated with Alexa 647-labeled avidin. Left: unhydrolyzed and non-biotinylated PET; middle: unhydrolyzed and biotinylated PET; right: hydrolyzed and biotinylated PET. Scale bar: 50 µm. (**B**) Mean Fluorescence (AU) of biotinylated PET discs with different spacer length exposed to Alexa-labeled avidin. Data are represented as mean ± SD, n = 3. (**C**) Schematic illustration of the avidin interaction with different biotinylated substrates.

Subsequently, the activated PET-NHS was functionalized with biotin. The activity of biotin immobilized on PET mesh was determined by immersing the mesh into a solution of avidin labeled with the Alexa Fluor 647 fluorescent dye. Unbound avidin was removed by rinsing the meshes, and the fluorescence intensity of the fiber was determined by fluorescent microscopy. The results demonstrated strong fluorescence in hydrolyzed biotinylated PET as compared to unhydrolyzed PET and unhydrolyzed biotinylated PET as control (Figure 3A), indicating high binding of avidin. The controls were prepared following a similar modification protocol as the hydrolyzed samples, but using unhydrolyzed meshes.

Since microsphere capture efficiency involves a balance between surface density of the capturing motif (biotin density) and molecular-level flexibility of the capturing motif [22], we varied the hydrolysis time to control the density of carboxylic acid groups on the PET fiber surface and introduced biotin conjugates bearing different spacer lengths and investigated the effect of these variables on avidin binding. Seven different hydrolysis time points between 0 to 4 h were utilized, and the molecular distance between the PET mesh surface and biotin was varied using two types of spacers: (i) ethylenediamine (EDA) and (ii) poly(ethylene glycol) (PEG). The coupling of EDA was achieved by reacting EDA on the NHS activated PET followed by addition of an NHS activated biotin (Figure 1A). The second coupling was done by reacting an amine-terminated PEG-biotin on NHS-activated PET surface. Within this set, two PEG-biotin conjugates were tested: a PEG dimer and a longer PEG octamer. The fluorescence of unfunctionalized PET (control) varied between three and six arbitrary units (AU) independent of the hydrolysis time (Figure 3B). While the fluorescence of PET-EDA-biotin meshes decreased for samples hydrolyzed longer than 0.5 h, PEG2-biotin had a constant maximal avidin binding at 15 AU, irrespective of time of hydrolysis. In contrast, PET-PEG_8_-biotin meshes reached a plateau at a minimal hydrolysis time of 1 h and a mean fluorescence of 30 AU. Taken together, PEG_8_-biotin was able to capture maximum avidin due to the introduction of a long-enough flexible spacer (Figure 3C) and thus was selected for subsequent studies.

### 2.3. Microsphere Capture under Turbulent Mixing

To demonstrate the capture of micrometer microspheres through orthogonal adhesion, PET-PEG_8_-biotin mesh was suspended by mounting on a cannula in a glass vial and was immersed in PBS containing a suspension of commercially available avidin-coated 10 µm microspheres. The microsphere suspension was then stirred at different rotational rates to investigate the microsphere attachment over a broad range of mixing conditions. The quality of the capture was assessed using optical microscopy to count the number of microspheres attached onto the mesh at different time points.

**Figure 4:**
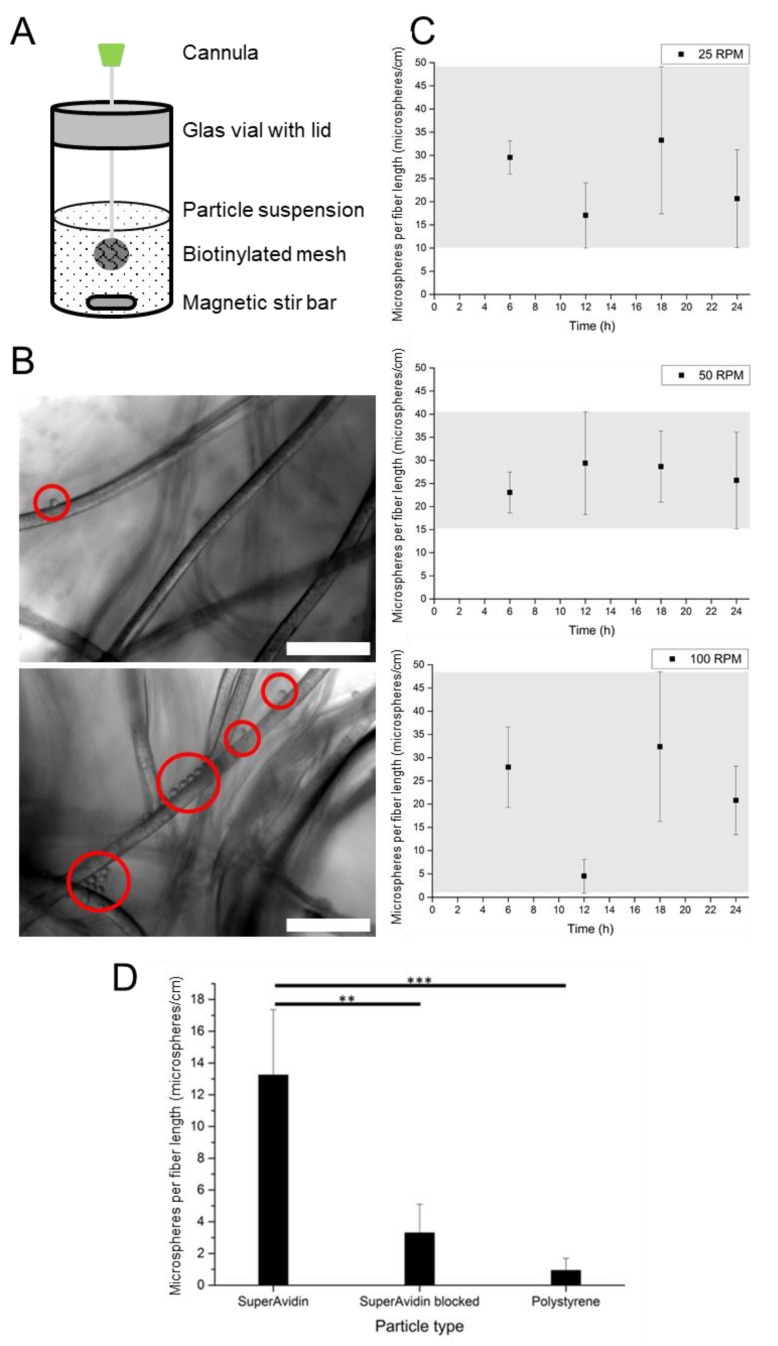
Microsphere retention under turbulent mixing. (**A**) Setup of the turbulent mixing experiment. (**B**) Microscope images of low (top) and high (bottom) microsphere attachment on modified PET meshes. Attached microspheres are circled in red. Scale bar: 100 µm. (**C**) Microsphere retention over time at different stirring conditions (25, 50, and 100 RPM). Data are represented as mean ± SD, n = 3. (**D**) Linear microsphere density (microspheres/fiber length) following turbulent mixing using polystyrene microspheres, avidin microspheres, and avidin-blocked microspheres. ** indicates significance (*p* < 0.01) between avidin and avidin blocked microspheres. *** indicates significance (*p* < 0.001) between avidin and polystyrene microspheres. Data are represented as mean ± SD, n = 3.

The mesh was immersed in a solution comprising 250,000 microspheres/mL, a density similar to cell densities in cell culture, for different periods (6, 12, 18, and 24 h) and the solution was stirred at 25, 50, and 100 RPM (Figure 4A). The speed of rotation was limited to 100 RPM to avoid vortexing of the suspension. After the designated time, the mesh was removed from the suspension, and the number of attached microspheres was quantified (Figure 4B). The attachment of microspheres on the fiber surface was influenced by the stirring speed. While the experiments stirred at 50 RPM generated reproducible and consistent results, stirring at 25 RPM and 100 RPM led to a broad distribution of captured microspheres (Figure 4C). At 50 RPM from 6 to 12 h, the number of captured microspheres plateaued at 29.4 ± 11.1 microspheres per cm of fiber length.

To confirm the specificity of the biotin capture, we performed a control experiment. The capture efficiency of the avidin microspheres was compared to two control conditions: (i) unmodified blank polystyrene microspheres and (ii) biotin-blocked avidin microspheres (avidin microsphere saturated with biotin before suspension). While the two negative controls showed marginal non-specific microsphere adhesion, binding of avidin microspheres on PET-PEG_8_-Biotin meshes was roughly 400% higher (Figure 4D). This demonstrates that the capture of the avidin coated microspheres is due to the biotin binding motif.

### 2.4. Microsphere Retention under Perfusion

**Figure 5:**
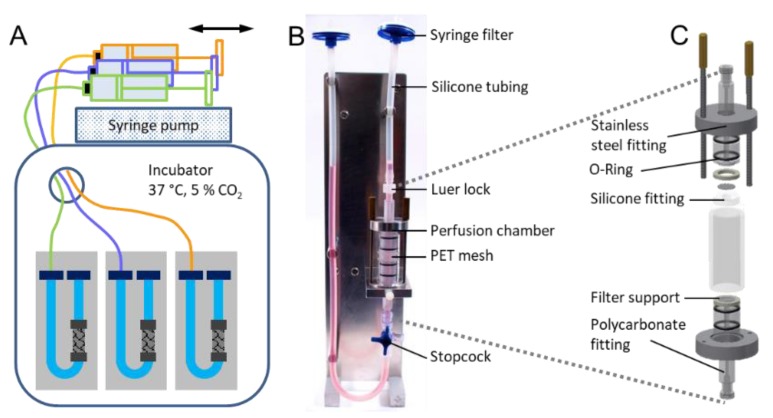
Microsphere retention using in-house developed perfusion bioreactor. (**A**) Schematic of the perfusion bioreactor setup. (**B**) Digital Photograph of the fully assembled perfusion bioreactor. (**C**) Exploded assembly drawing of the perfusion chamber of the bioreactor.

Having demonstrated that the PET-PEG_8_-biotin mesh can successfully capture avidin microspheres under turbulent mixing, next, the efficiency of the system to capture microspheres under perfusion, which simulates the circulation of micron-scale objects such as cells was tested. A custom-built set of U-shaped perfusion bioreactors were placed in a biological incubator (37 °C, 5% CO_2_) and connected to a syringe pump perfusing the liquid at 0.2 mL/s in an oscillating mode (Figure 5A,B). The PET-PEG_8_-biotin meshes were placed in the perfusion chamber, between the two o-rings as shown in Figure 5C. The system was injected with 4 mL of a 250,000 microspheres/mL suspension which was perfused for 18 h.

**Figure 6:**
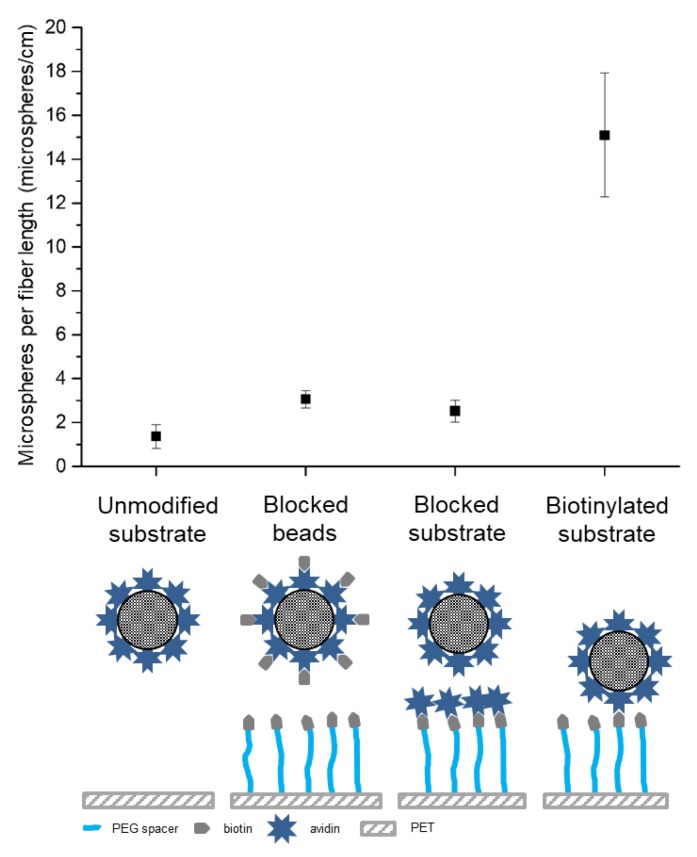
Microsphere attachment on PET surfaces using a dynamic perfusion bioreactor. Data are represented as mean ± SD, n = 3.

For control experiments, we used unmodified PET mesh, avidin-blocked PET-PEG_8_-biotin meshes, and biotin-blocked microspheres. Results demonstrated that PET-PEG_8_-biotin could capture 15.1 ± 2.8 microspheres/cm as compared to control meshes, which captured between 1.4 ± 0.5 and 3.1 ± 0.4 microspheres per cm of fiber (Figure 6), thus demonstrating the specific capture of avidin-coated microspheres on biotinylated PET substrates under constant flow.

## 3. Discussion

Selective capturing platforms for low abundance microscopic objects such as low occurring cell populations or circulating tumor cells are gaining importance in diagnostics and biomarker development. While physical capture of cells might be associated with a non-specific selection, biochemical capture might modulate the cell behavior following immobilization. In this study, we designed a capturing method based on biorthogonal binding. As a proof of concept, biotinylated PET meshes were used for the capture of avidin functionalized microspheres.

Mild hydrolysis of PET surgical mesh yielded a COOH modified surface. The diluted NaOH solution cleaved the ester bonds of the polymer, thus increasing the amount of accessible COOH groups on the mesh surface. However, as previously reported in the literature [23,24,25], this technique led to a mild PET surface modification as shown on the SEM images in this study. Therefore, we optimized the reaction time to minimize fibers surface modification. The observed changes in accessible COOH surface concentration over time during hydrolysis can be attributed to the surface modification. Further hydrolysis of the PET surfaces after reaching the maximum accessible COOH concentration resulted in a drop in the concentration of this functional group, suggesting that the top layer of the PET fibers had likely peeled off, thus exposing a new unhydrolyzed polymer layer with a lower COOH concentration (Figure 1B). This was also confirmed by the pitted fiber surface observed on SEM images (Figure 2). As the hydrolysis resumes, a new polymer layer is hydrolyzed, and the COOH concentration increases again. The drop in accessible COOH concentration again accompanies PET fibers surface modification of the next layer. With this precise study, we can tailor the hydrolysis process to reach accessible COOH concentration of choice. Following this, COOH groups were activated into ester groups that can react with nucleophilic functional groups such as amines allowing for the functionalization of the PET mesh with biotin.

It has been reported that the binding pocket for biotin is 0.3 nm from the protein surface [26,27]. Therefore, we hypothesized that the length between biotin and the PET surface would affect the binding of the PET-biotin meshes to avidin. Indeed, we observed a relationship between the binding efficiency and the distance of biotin from the PET surface. We used the following three different length estimates, ~0.37 nm for ethylenediamine [28]—calculated from water computed conformation—0.6 nm for the PEG_2_ and 2.25 nm for PEG_8_ [29]. This relation was attributed to the reduced availability of biotin binding with the short spacer. Further, avidin is a large protein (66 kDa) with four binding pockets. If bound closely to the surface of PET mesh; an avidin molecule once captured will block a wide surface area of approximately 20 nm^2^ thereby leading to steric hindrance and would also limit the attachment of other avidin molecules to the adjacent biotin proteins [30]. Furthermore, a short spacer limits the movement of PET-immobilized biotin that in turn affects the accessibility of avidin to the biotin-binding pocket [31]. In contrast, we showed that longer PEG spacer consisting of 8 monomer units allow the biotin to be flexible above the PET surface, thus improving the accessibility of (strept)avidin to the biotin-binding pockets. Hence, we demonstrated that allowing a certain degree of movement to biotin on PET surface increases the biotin-avidin interaction and therefore the capture of avidin-tagged objects. This interaction is independent of the hydrolysis of the PET, i.e., accessible COOH density and of the density of biotin immobilized on the surface. This result suggests that due to the size of the biotin-avidin complex and its coupling mechanism, increasing the biotin density on the PET surface above a certain threshold does not lead to an increase in avidin coupling. The capture density is therefore strictly limited by the degree of movement allowed to biotin to find the right conformation to bind to avidin.

With this optimized biotin functionalized PET mesh, the capture efficacy of 10 µm avidin-modified microspheres suspension was assessed. Under turbulent mixing, we observed that most microspheres were captured at 50 RPM; samples incubated at 25 RPM and 100 RPM were exposed to mild or drastic mixing respectively and led to a non-uniform capture of microspheres. Therefore, for efficient capture of microspheres, the mixing flow rate needs to be optimized to maximize the interaction between the microspheres and the mesh. Slow rates limit the contact between the microsphere and the mesh, and severe mixing conditions reduce the contact times for binding [32,33]. Although the avidin-biotin interaction is reversible [34], the reduced number of immobilized beads observed in both 50 RPM and 100 RPM might not originate from a shear stress induced rupture of the avidin-biotin complex. Hence, identification of the optimal mixing speed for the capture of microspheres is the key towards the development of a mesh that yields high attachment rates.

Combination of optimized mixing conditions and PET-PEG_8_-biotin mesh could be applied for the capture of circulating cells such as avidin-expressing mammalian cells, in vivo [15]. As a proof of concept, 10 µm avidin-coated microspheres were perfused through our custom-made bioreactor. The velocity was chosen based on a previous study that demonstrated that this perfusion rate leads to fewer cell attachment on the walls of the bioreactor or settlement on the base of the bioreactor [35]. In this study, we were able to demonstrate that PET-PEG_8_-biotin mesh specifically captures avidin microspheres under in vivo-relevant conditions. The observed attachment rates can be used to calculate a theoretical capture efficiency of this system for living cells. As an example, the diameter of mouse L929 fibroblast cells attached to untreated PET surfaces varies between approximately 10 µm and 50 µm as a function of cell spreading [36]. Thus, an attachment rate of 29 microspheres/cm in our system would translate into coverage of 2.9–14.5% of the fiber length with attached cells.

In sum, the results of this study demonstrate the successful use of biotin-avidin coupling chemistry for the capture of microspheres under physiologically relevant conditions. These results could be translated for the capture of avidin expressing cells. As demonstrated by our capture study under mixing, genetically modified cells could be immobilized with a similar biotin-PET meshes in a bioreactor and avidin expressing circulating cells could be captured under flow as suggested by our results in the dynamic perfusion bioreactor.

## 4. Materials and Methods

### 4.1. Materials

Poly(ethylene terephthalate) (PET) surgical meshes (PETNF203) were purchased from Textile Development Associates, Inc. (Brookfield, CT, USA). Avidin-coated microspheres (CP01N) were purchased from Bangs Laboratories, Inc. (Fishers, IN, USA), sulfosuccinimidyl-4-*O*-(4,4-dimethoxytrityl) butyrate (Sulfo-SDTB) was obtained from bioWORLD (Dublin, OH, USA). Biotin N-hydroxysuccinimide ester was purchased from AppliChem (Darmstadt, Germany). Amine-terminated poly(ethylene glycol) dimer functionalized with biotin (Amine-PEG_2_-Biotin) and the Alexa Fluor 647 Microscale Protein labeling kit were purchased from Thermo Fisher Scientific (Dreieich, Germany). All other chemicals were purchased from Sigma-Aldrich (Darmstadt, Germany). Chemical reagents and solvents used for the synthesis were of analytical grade and used as received. All synthesis experiments were carried out in air. Water was deionized with a Milli-Q Reference water purification system manufactured by Millipore Corporation (Darmstadt, Germany). All reactions involving the modification of PET were carried out in a Snycore^®®^ Reactor R-24 (Büchi, Flawil, Switzerland). Scanning electron microscopy (SEM) was performed on a Quanta 250 FEG microscope using an accelerating voltage of 20 kV in high vacuum from FEI (Hillsboro, OR, USA). The micrographs were then analyzed with xT Microscope Control (4.1.4).

### 4.2. Statistical Analysis

Statistical evaluation was done using the non-parametric Mann-Whitney test with the software BrightStat [37]. A probability of *p* ≤ 0.05 was considered as significant. Significance is notated with asterisks as follows: * *p* ≤ 0.05, ** *p* ≤ 0.01, and *** *p* ≤ 0.001. If not stated otherwise, values are reported as mean ± standard deviation.

### 4.3. Hydrolysis of PET

Discs with a diameter of 4.00 mm (average weight: 2.50 mg) were cut out from a non-woven PET mesh using a biopsy punch. PET discs were then placed in a scouring solution (2 mL/disc; 1% (*v*/*v*) Tween 20 in deionized water) and stirred (300 RPM) at 60 °C for 35 min. The scouring solution was then discarded, the discs blotted dry on paper tissue, and washed with deionized water (3 × 5 mL/disc). Afterward, the discs were blotted dry again. In adaptation to Bide et al. [10], the PET discs were hydrolyzed in an aqueous NaOH solution (2.00 mL/disc; 0.5% (*w*/*v*)) for different durations (up to 12 h) at 98 °C, 300 RPM, and under atmospheric pressure. Before the heating started, the reaction mixture was set under vacuum for 3 min to allow thorough wetting of the disc. Following hydrolysis, the discs were blotted dry and washed with deionized water (3 × 5 mL/disc). Subsequently, the discs were blotted dry again.

### 4.4. Functionalization of PET

Hydrolyzed PET mesh (PET-COOH, 1 eq.) was activated in 2-(*N*-morpholino)ethanesulfonic acid (MES) buffer (2.00 mL, pH 5.40) and then 1-ethyl-3-(3-dimethylaminopropyl)carbodiimide (EDC) solution (12.7 µL, 10.0 mM in MES, 127 nmol, 1000 eq.) and N-hydroxysuccinimide (NHS) solution (12.7 µL, 10.0 mM in MES, 127 nmol, 1000 eq.) were subsequently added. Initially, the reaction mixture was set under vacuum to allow thorough wetting of the disc and then was shaken (300 RPM) under atmospheric pressure at room temperature (RT) for 60 min. The activated disc was washed with PBS buffer (3 × 5 mL), blotted dry again and placed in phosphate buffered saline (PBS) buffer (2 mL, pH 7.40) for further functionalization. To this PET disc (PET-NHS, 1 eq.), *O*-(2-aminoethyl)-*O*′-[2-(biotinylamino)ethyl]octaethyl glycol solution (12.7 µL, 10.0 mM in PBS, 127 nmol, 1000 eq., resulting in PET-PEG_8_-Biotin), Amine-PEG_2_-Biotin solution (12.7 µL, 10.0 mM in PBS, 127 nmol, 1000 eq., resulting in PET-PEG_2_-Biotin) or ethylenediamine (EDA) solution (12.7 µL, 10.0 mM in PBS, 127 nmol, 1000 eq., resulting in PET-EDA) was added. The reaction mixture was set under vacuum for 3 min and then, under atmospheric pressure, shaken (300 RPM) for 120 min at RT. The meshes were washed with PBS and blotted dry for further experiments.

Further biotinylation of PET-EDA was accomplished by placing a disc (PET-EDA, 1 eq.) in PBS (2.00 mL, pH 7.40) followed by addition of (+)-biotin N-hydroxysuccinimide ester (100 µg, 293 nmol, 2300 eq., resulting in PET-EDA-Biotin) dissolved in PBS (200 µL). The reaction mixture was set under vacuum for 3 min, then under atmospheric pressure and stirred (300 RPM) for 120 min at RT. The disc was washed with PBS and blotted dry for further experiments.

### 4.5. Determination of Accessible COOH Concentration on PET Surface

The colorimetric Sulfo-SDTB method was used to determine the amount of immobilized amine. This was used to indirectly calculate the accessible COOH concentration on the fiber surface after hydrolysis or on solid surfaces in general. When treated with perchloric acid, Sulfo-SDTB releases a trityl cation which strongly absorbs visible light (ε = 70,000 1/(M cm)). The colorimetric assay was adapted from Gaur and Gupta [17], following a protocol described elsewhere [38].

A PET-EDA disc (16 mm diameter and known weight, treated accordingly to the small discs) was soaked in the Sulfo-SDTB solution for 10 min at RT, then washed with deionized water (3 × 20.0 mL) to remove excess Sulfo-SDTB and blotted dry. The cleavage of the trityl cation was induced by placing the disc in perchloric acid followed by mixing in order to get an orange-colored solution. The absorbance was then measured at 494 nm using a plate reader (Biotek, Winooski, VT, USA), compared to a standard curve, and normalized to the calculated surface area of the PET disc.

### 4.6. Alexa Fluor 647 Labeling of Avidin (Avidin-Alexa)

The labeling of avidin with Alexa Fluor 647 was carried out following the detailed instructions in the protocol provided by the manufacturer. Briefly, an aqueous avidin stock solution (5.00 mg/mL) was prepared. 40.0 µl of this stock solution was diluted with 50.0 µL of deionized water and 10.0 µL of bicarbonate buffer (1 M, Component B of the labeling kit). Afterward, 10.0 µL of deionized water was added to the vial of Alexa Fluor 647 succinimidyl ester (Component A of the labeling kit), resulting in a reactive dye solution with a concentration of 7.94 nmol/µL. Then, 5.00 µL of this stock solution was added to the avidin-containing solution, followed by mixing and incubation for 15 min at RT. Purification of the labeled avidin was performed using a spin column filled with a resin. The final concentration of labeled avidin in solution was 0.940 mg/mL as determined on a NanoDrop spectrophotometer (Thermo Fisher Scientific, Dreieich, Germany). The solution was stored at 4 °C until further use.

### 4.7. Labeling of PET with Avidin-Alexa

Before use, Alexa 647-labeled avidin solution was diluted one hundred times with PBS containing PSG (penicillin, streptomycin, glutamine) and HAS (human serum albumin) (both 1% (*v*/*v*)). A small piece of PET mesh (PET-COOH, PET-EDA-Biotin, PET-PEG_2_-Biotin or PET-PEG_8_-Biotin, approx. 0.250 mg) was placed in HSA (100 µL, 3%) for 1 h to block the surface of the fibers to avoid non-specific absorption of avidin. After removal of the HSA, the meshes were incubated in the diluted Avidin-Alexa solution (50.0 µL) overnight at RT. The reactive dye solution was then removed, and the meshes were washed with PBS (3 × 100 µL). Several fibers were removed from the mesh, and the amount of Avidin-Alexa on the fiber surface was measured using a fluorescence microscope (exposure time: 2217 ms, excitation: 560 nm, filter: 625 nm, light source: LED). Mean fluorescence of single fibers was determined using ImageJ [39].

### 4.8. Capture of Microspheres under Turbulent Mixing

A biotinylated PET disc (PET-PEG-Biotin) was mounted on a cannula which was then placed in a glass beaker filled with an avidin-coated microsphere suspension (5.00 mL, 250,000 microspheres/mL). The suspension was stirred with a magnetic stirrer at 25, 50 and 100 RPM. The mesh was removed from the suspension after the intended time period (varied between 6 h and 48 h) and imaged at 10 different spots with images in three different focal planes at each spot within a single disc. These images were analyzed using ImageJ, and the density of captured microsphere was expressed as the number of microsphere per unit of fiber length. Only microspheres aligned parallel to the fiber were counted as attached microspheres.

### 4.9. Design of the Perfusion Bioreactor

A perfusion bioreactor was designed with a U-shaped system centered around a chamber that holds the PET meshes while allowing continuous perfusion of liquids through the scaffolds in alternate directions (Figure 5). The chamber comprises two stainless steel fittings and two polycarbonate fittings screwed to each other. The two sets were then each connected to two O-rings, a stainless steel filter support and a silicone fitting. The silicone fitting in the center of the chamber held the PET discs. The whole assembly was then fitted in a glass cylinder. Following the fitting of the chamber assembly, the top and bottom stainless steel fittings were tightened using two screws that pass through the whole assembly. The entries of the chamber were connected to a small and a long silicone tubings. The smaller silicone tubing was connected to the top of the chamber through a Luer lock, and the longer silicone tubing was connected to the bottom of the chamber through a Luer lock and three-way stopcock. The longer silicone tubing was then fitted onto an assembly holder to form the U-shape. To prevent any contamination, the ends of the both silicone tubings were connected to a 0.2 µm syringe filter. The filter on the longer silicone tubing was then connected to a syringe pump to create the dynamic flow.

### 4.10. Capture of Microspheres under Perfusion

A biotinylated PET disc (PET-PEG-Biotin) was mounted in the preassembled perfusion bioreactor, which was then injected with an avidin-coated microsphere suspension (4.00 mL, 250,000 microspheres/mL). The bioreactor was placed in an incubator (37 °C, 5% CO_2_) and connected to a syringe pump. Perfusion was performed with a velocity of 0.2 mL/s for 18 h. Following this, the discs were removed from the bioreactor and imaged as mentioned in Section 4.8. The images were analyzed using ImageJ, and the density of microsphere attachment was expressed as a number of microspheres per unit of fiber length. Only microspheres aligned parallel to the fiber were accounted as attached microspheres.

## 5. Conclusions

This work aimed to demonstrate the capture of microspheres in the size range of mammalian cells on polymer fibers using avidin-biotin interaction. We demonstrate that non-woven PET meshes can be mildly hydrolyzed using a dilute alkaline solution to generate COOH groups on the fiber surface. Subsequent functionalization of the meshes by activation of the COOH groups using EDC/NHS chemistry allowed the biotin to be immobilized at different distances from the PET surface. We demonstrated that biotin linked through longer PEG octamer spacer was more efficient in the capture of avidin-coated microspheres as compared to PEG dimer. The affinity of the avidin-biotin system was shown to be sufficient for the immobilization of cell-shaped microspheres under vigorous mixing conditions as well as under perfusion flow. These results demonstrate the possibility of using the PET-PEG_8_-biotin system to capture micrometer-sized objects and represent a significant step towards synthetic orthogonal capture of cells under perfusion or shear flow in subsequent studies.
